# Revealing thermally-activated nucleation pathways of diffusionless solid-to-solid transition

**DOI:** 10.1038/s41467-021-24256-9

**Published:** 2021-06-30

**Authors:** Minhuan Li, Zhengyuan Yue, Yanshuang Chen, Hua Tong, Hajime Tanaka, Peng Tan

**Affiliations:** 1 State Key Laboratory of Surface Physics and Department of Physics, Fudan University, Shanghai, China; 2 School of Physics and Astronomy, Shanghai Jiao Tong University, Shanghai, China; 3 Department of Physics, University of Science and Technology of China, Hefei, China; 4 Department of Fundamental Engineering, Institute of Industrial Science, University of Tokyo, Tokyo, Japan; 5 Research Center for Advanced Science and Technology, University of Tokyo, Tokyo, Japan

**Keywords:** Phase transitions and critical phenomena, Structure of solids and liquids, Colloids, Phase transitions and critical phenomena, Structure of solids and liquids

## Abstract

Solid-to-solid transitions usually occur via athermal nucleation pathways on pre-existing defects due to immense strain energy. However, the extent to which athermal nucleation persists under low strain energy comparable to the interface energy, and whether thermally-activated nucleation is still possible are mostly unknown. To address these questions, the microscopic observation of the transformation dynamics is a prerequisite. Using a charged colloidal system that allows the triggering of an fcc-to-bcc transition while enabling in-situ single-particle-level observation, we experimentally find both athermal and thermally-activated pathways controlled by the softness of the parent crystal. In particular, we reveal three new transition pathways: ingrain homogeneous nucleation driven by spontaneous dislocation generation, heterogeneous nucleation assisted by premelting grain boundaries, and wall-assisted growth. Our findings reveal the physical principles behind the system-dependent pathway selection and shed light on the control of solid-to-solid transitions through the parent phase’s softness and defect landscape.

## Abstract (web summary)

Normally the diffusionless solid-to-solid transition between phases are driven by athermal processes, due to strain being overwhelmingly dominant. Here, the authors present a unique series of in-situ particle level observations of the solid-to-solid transition in colloidal particles suspended in a solvent, revealing new transition pathways.

## Introduction

Diffusionless solid-to-solid transitions (martensitic transition) have been widely observed in both hard materials, such as metals, alloys, and ceramics^1–10^, and soft-matter systems, such as protein solutions and colloidal suspensions^11–14^. Despite the variety of these systems with various softness and symmetry^4–14^, one common intriguing feature is the formation of rich microstructures via complex nucleation and growth pathways. Nuclei formation has so far been believed to be athermal because immense strain energy is required to overcome a high barrier, which is the case for the steel’s martensitic transition. This means that the process must be initiated from defect-stabilised nuclei already existing in the parent solid well above the transformation temperature. The subsequent growth of the product solid is then governed by a delicate balance between elastic, interfacial, and chemical energies^1^.

However, such a scenario may no longer apply to soft materials in which the lattice is easy to deform because thermal activation cannot be neglected anymore. Nonetheless, to what extent the athermal nucleation persists as the strain energy reduces and whether there is a new realm of nucleation behaviours, e.g. thermally activated nucleation and interface energy-dominated pathways, remains mostly unknown. To fully understand the kinetics of solid-to-solid transformation, it is critical to elucidate softness-dependent nucleation behaviours. Understanding these processes will have a considerable impact on the microstructure control of numerous soft materials.

Martensitic transitions have been experimentally observed through X-ray scattering^15^, acoustic emission^16^, transmission electron microscopy^17^ and calorimetry^18^. However, these techniques can rarely access the single-particle-level nucleation kinetics of martensitic transitions. Fortunately, as an important class of soft materials, colloidal suspensions are suitable for real-space three-dimensional (3D) microscopy observations. The controllability of the size, shape, and interparticle interaction of colloidal particles has provided deep microscopic insights into various phase transitions, including crystallisation^19–22^, melting^23,24^, glass transition^25^ as well as solid-to-solid transitions. Concerning martensitic transitions in colloidal crystals, many studies have been performed by applying an electric field^12,26^, changing the particle shape, confinement or volume fractions^14,27–32^, shear^33,34^ and adjusting the DNA grafting^13,35^. These studies have confirmed the diffusionless nature of the transition, as in hard materials.

However, even with colloidal particles, it has been quite challenging to experimentally access the microscopic kinetics in the nucleation stage due to the difficulty in a non-perturbative initiation of the transition while performing in-situ 3D confocal microscopy observations. Accordingly, the following two critical issues concerning the martensitic transition kinetics in soft materials have so far remained largely unexplored: (1) To what extent does the soft nature of materials affect the nucleation kinetics? (2) How does the system choose appropriate nucleation pathways to overcome the free-energy barrier? Here, we address these fundamental questions experimentally with a unique ion-exchange method that can initiate an fcc-to-bcc transition in a non-perturbative manner. We find both athermal and thermally activated pathways controlled by the softness of the parent crystal. In particular, we reveal three new transition pathways: the in-grain homogeneous nucleation driven by spontaneous dislocation generation, the heterogeneous nucleation assisted by premelting grain boundaries, and the wall-assisted growth.

## Results

### Experiments and methods

**Fig. 1:**
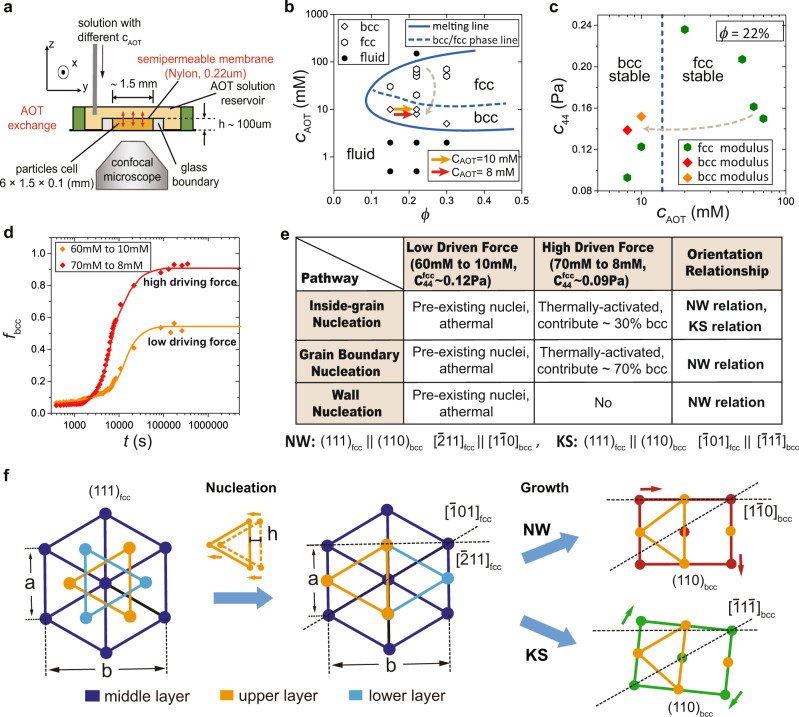
Experiment details and softness-dependent pathways. **a** The side view of our experimental setup. The sample cell contains charged PMMA colloidal particles suspended in a density- and refractive index-matched solvent, covered by a semi-permeable membrane with a pore size of 0.22 μm. Ionic AOT micelles with the size of ~1 nm can pass through the membrane, whereas PMMA particles (*σ* ~ 1.8 μm) cannot. We first form fcc crystals at a high *C*_AOT_ (fcc-stable), then gently change the AOT solution in the reservoir by replacing the one with low *C*_AOT_ (bcc-stable). Quick ion exchange (about 10 s) triggers an fcc-to-bcc transition inside the sample cell under confocal microscopy observation. **b**, Equilibrium phase diagram as a function of the particle volume fraction *ϕ* and the AOT concentration *C*_AOT_. We perform experiments at *ϕ* ~ 22%. We illustrate two typical cases of the fcc-to-bcc martensitic transitions from 60 mM AOT to 10 mM (see the orange arrow, weak-driving force) and from 70 to 8 mM (see the red arrow, stronger driving force). **c** The *C*_AOT_-dependence of the shear modulus component *C*_44_ for parent fcc lattice (${C}_{44}^{{\rm{fcc}}}$) and final bcc lattice (${C}_{44}^{{\rm{bcc}}}$). Note the very low value of ${C}_{44}^{{\rm{fcc}}}$ at *C*_AOT_ = 8 mM. The blue verticle dotted line indices the fcc–bcc phase line. **d** The bcc solid fraction *f*_bcc_ as a function of time *t* after the solution exchange. *t* = 0 represents the time when we start to observe (~60 s after changing *C*_AOT_). For the low-modulus, strong-driving-force transition (*C*_AOT_ ~ 8 mM), the bcc solids fraction increases up to more than 90% of the system, whereas for the weak-driving-force transition (*C*_AOT_ ~ 10 mM), it saturates around 50%. **e** The summary of the kinetic pathways of the fcc-to-bcc transitions observed in our system. The pathways can be classified into two classes (athermal and thermally activated). In a high driving force system, the GB-assisted pathway has a larger contribution to bcc crystallites (70%) than the in-grain homogeneous pathway (30%), which are estimated by investigating the formation of bcc crystallites in randomly selected regions of a sample when the amount of bcc solids reaches about 40–50% of the system. This timing is in the growth stage after the nucleation stage, but bcc solids formed by the in-grain nucleation and GB-assisted nucleation can still be distinguished since it is before they merge. **f** The two-step creation process of the NW/KS relationship. First, the upper and lower layers of fcc (111) plane slip relative to the middle layer at the initial nucleation stage (see the inter-layer sliding *h*). Then, during further development, two types of intra-layer deformation (see the change of *b*/*a*) lead to the NW and KS relationships.

To access the kinetic process of solid-solid transition microscopically, we have designed a new experimental setup using a charged colloidal system, which allows us to control the parent fcc crystal’s softness and probe its influence on the kinetics of fcc-to-bcc transitions at a single-particle-level resolution. Specifically, we use charged PMMA (polymethyl methacrylate) colloidal particles (diameter *σ* ~ 1.8 μm, dyed with nitrobenzoxadiazole) suspended in a density- and refractive index-matched solvent. The interparticle interaction can be effectively described by the hard-core repulsive Yukawa potential: $u(r)=\alpha \exp [-\kappa \sigma (r/\sigma -1)]/(r/\sigma )$ for *r* > *σ*, with 1/*κ* being the Debye screening length in the suspension and *α* being a parameter of the interaction strength. 1/*κ* and *α* can be adjusted by the ionic AOT (sodium di-2-ethylhexyl sulfosuccinate) surfactant concentration, *C*_AOT_^36–38^. We employ a special ion-exchange experimental protocol (see Fig. 1a) that allows us to change the interparticle interaction, *u*(*r*), quickly by adjusting *C*_AOT_ in the reservoir. We trigger the fcc-to-bcc transition at various *C*_AOT_ and observe the kinetics in-situ using a Leica SP8 fast confocal microscope. This method enables us to follow the microscopic kinetics of martensitic transitions with a single-particle-level resolution from the very beginning (see ‘Methods’ on the details of experiments).

**Fig. 2:**
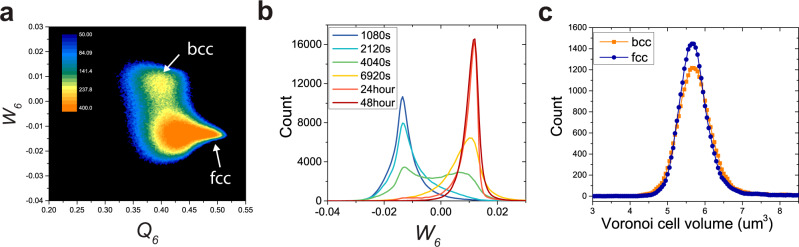
Illustration of the fcc-to-bcc transition in the 2D order-parameter space. **a** 2D distribution of particles' local order in the *Q*_6_ − *W*_6_ parameter space for the strong-driving force (the data accumulated from *t* = 0 s to *t* = 8000 s). From the fcc-solid region of high *Q*_6_ with negative *W*_6_, the particles undergo the decrease in *Q*_6_ and the increase in *W*_6_ on the 2D parameter space and eventually transform to bcc solid with a jump of *W*_6_. We can see that liquid particles (*Q*_6_ < 0.35) rarely participate in the transformation process. **b** The *W*_6_ distribution of particles at different time. It suggests the effectiveness of *W*_6_ to distinguish fcc from bcc. **c** The Voronoi cell volume, which is inversely proportional to the local density, for fcc particles (the parent phase) and bcc particles (the product phase). We can see a tiny density difference (0.68%) between the two phases.

We use two structural order parameters, which are coarse-grained bond orientational order parameters, *W*_6_ and *Q*_6_, to quantify the single-particle-level local structures (see Methods on their definitions). *W*_6_ is used to distinguish fcc and bcc by its sign (*W*_6_ ≥ 0 for bcc and *W*_6_ < 0 for fcc), whereas *Q*_6_ quantifies the degree of crystalline order of the solid (particles with *Q*_6_ < 0.35) are regarded as defective structures, which mainly consist of highly distorted (defective) solids with a small amount of liquid-like (*Q*_6_ < 0.25) particles^21,22,39–41^ (Fig. 2).

### Softness-dependent nucleation behaviours

In general, the nucleation barrier for the formation of the nucleus of the product phase can be written as^1,31^:

1 $${{\Delta }}G=-V\rho {{\Delta }}\mu +A\gamma +{E}_{{\rm{strain}}}-{E}_{{\rm{defect}}},$$

where *V* is the nucleus volume of the product phase, *ρ* is the particle number density of the product nuclei, Δ*μ* (>0) is the chemical-potential difference between the parent and product phases, *A* is the nucleus surface area, *γ* is the surface tension, *E*_strain_ is the extra misfit strain energy induced by the volume difference between the two phases that depends on the elastic modulus of the parent phase, and *E*_defect_ is the energy of pre-existing defects per volume *V*. The first term on the right-hand side is a driving force of the transition. The second and third terms represent the surface- and strain-energy costs of the product phase’s nucleation. In hard materials, *E*_strain_ is usually quite large, and the nucleation barrier is much larger than the thermal energy, *k*_*B*_*T*. Accordingly, internal or applied stress is the major controlling factor of nuclei generation. We refer to this case as athermal nucleation. In such a case, pre-existing defects promote the nucleation because defective structures have low local modulus and significantly reduce *E*_strain_. It was phenomenologically shown for athermal nucleation behaviours in steels that nuclei stabilised by stresses already pre-exist in the parent phase, and their growth is governed by the balance between *E*_strain_, *Aγ*, and Δ*μ*. In contrast, the much smaller *E*_strain_ in soft materials than hard materials can provide other nucleation pathways to reduce the nucleation barrier, depending on the condition. They include spontaneous nucleation with and without applied stresses and pathways controlled by the interface energy *Aγ*. Phenomenologically, thermally driven nucleation could happen with or without applied stresses. For example, a previous study of colloidal thin films revealed a nucleation behaviour triggered by the internal fluid flow, which can be regarded as stress-induced spontaneous nucleation^14^.

In our system with a particle volume fraction of *ϕ* ~ 22%, the fcc–bcc phase-transition line locates near *C*_AOT_ ~ 15 mM, and the bcc melting line locates near *C*_AOT_ ~ 6 mM, as shown by the phase diagram in Fig. 1b. A rapid decrease of *C*_AOT_ from a high concentration (fcc-stable, i.e. *C*_AOT_ = 70 mM) to a low concentration (bcc-stable, i.e. *C*_AOT_ = 10 mM) provides the system with a driving force Δ*μ* of the fcc-to-bcc transition. At the same time, reducing *C*_AOT_ (below 15 mM) softens the parent fcc lattice, as shown by the *C*_AOT_-dependence of the shear modulus component ${C}_{44}^{{\rm{fcc}}}$ and ${C}_{44}^{{\rm{bcc}}}$ in Fig. 1c (see ‘Methods’ for *C*_44_ definition and measurements). We can use this property to probe how the lattice softness affects the microscopic kinetics. At each *C*_AOT_, we estimate the effective temperature *T* in units of the melting temperature *T*_*m*_ of the bcc lattice, using the Lindemann criteria: *T* ~ *δL*^2^(*C*_AOT_)*T*_m_/*δL*^2^(*C*_m_), where *C*_m_ is the AOT concentration close to the bcc melting line at *ϕ* ~ 22%.

Typically, a rapid jump from *C*_AOT_ = 60 mM, where fcc is stable, but a small amount of pre-existing bcc exist, to *C*_AOT_ = 10 mM (indicated by the orange arrow in Fig. 1b), where bcc is stable, produces a weak-driving force of the transition at *T* ~ 0.65*T*_m_ (10 mM). On the other hand, a rapid jump from *C*_AOT_ = 70 mM (fcc-stable, pre-existing bcc is less frequent than at *C*_AOT_ = 60 mM) to *C*_AOT_ = 8 mM (see the red arrow in Fig. 1b) produces a stronger driving force (larger Δ*μ*) but leads to a lower ${C}_{44}^{{\rm{fcc}}}$. A strong-driving-force and low-modulus thus characterise the resulting transition at *T* ~ 0.9*T*_m_ (8 mM). Careful observations reveal a variety of dynamics of the fcc-to-bcc transition, depending upon ${C}_{44}^{{\rm{fcc}}}$ and the degree and type of defects in the initial parent fcc crystal (i.e. defect-free, grain boundaries, and boundary walls).

First, we can notice that the fraction of the produced bcc solids, *f*_bcc_, depends on ${C}_{44}^{{\rm{fcc}}}/{{\Delta }}\mu$, as shown in Fig. 1d. The shape of the temporal change of *f*_bcc_ is also different between the two cases. The low-modulus, the strong-driving-force condition makes the growth of *f*_bcc_ more abrupt. On the other hand, the time taken to reach the final saturation of the bcc solids fraction is similar between the two cases (~23 h). It implies that the final bcc solids fraction is determined by the number of nucleation sites already existing in the initial parent phase^1,7^.

We also find that a system selects the optimal nucleation pathway, depending on the conditions, as summarised in Fig. 1e. For weak-driving-force cases, the athermal nucleation behaviours are dominant. They include the three pathways observed at 10 mM, which are initiated from pre-existing bcc nuclei at different sites in the system (inside the fcc grains, on the grain boundaries, and at the flat wall surface). In these cases, the bcc nuclei are already pre-existing in the parent fcc lattice at *C*_AOT_ = 60 mM, specifically at the triple junction of grain boundaries and at the flat walls surfaces, and their growth is mainly governed by the balance between *Aγ*, and Δ*μ*. These behaviours are much consistent with the athermal features observed in hard materials, such as metals and alloys. On the other hand, surprisingly, we discover that the nucleation behaviour changes from athermal to thermally activated when the parent fcc lattice becomes soft enough (see the two pathways at 8 mM): spontaneous nucleation of new bcc crystallites are observed inside the fcc parent lattice and near the premelting grain boundaries. For these two pathways, we find that the flow in the sample during the nucleation (measured by a particle tracking of 20 s that is about ten times longer than the particles’ Brownian time) is negligible (<2 nm/s), indicating that the pathways belong to thermally activated nucleation in the absence of applied stresses.

**Fig. 3:**
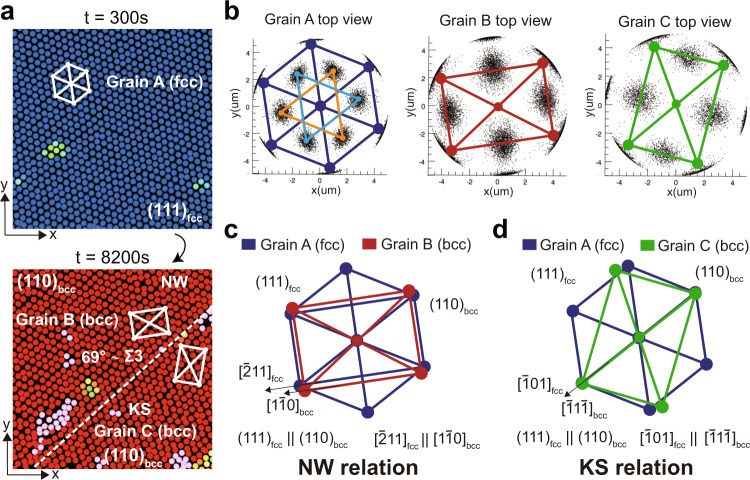
Examples of bcc twinning grains in the product bcc solid. **a** Creation of the bcc twinning grains B and C from fcc grain A (the inside-grain nucleation). Colour represents the value of *W*_6_. Pink particles are defect clusters with *Q*_6_ < 0.35. **b** Representation of the orientational relationship by the bond orientational diagram of each grain. **c** The NW relationship between fcc grain A and bcc grain B. **d** The KS relationship between fcc grain A and bcc grain C. The twinning boundary between grains B and C forms with a tiny angle rotation of the grains.

**Fig. 4:**
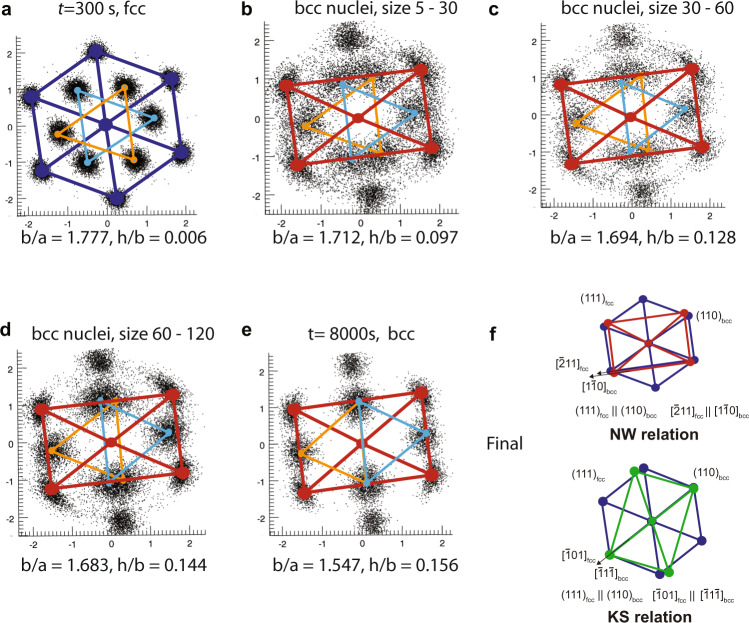
The creation kinetics of NW/KS relationship. Scatter plots are 2D projections of Bond Orientation Order Diagrams (BOODs) of local nuclei. Spheres represent centres of clusters on BOODs, viewed as the averaged lattice structures. Two geometry metrics *b*/*a* and *h*/*b* are calculated from these averaged lattices, illustrating the transition from fcc (*b*/*a* = 1.73, *h*/*b* = 0) to bcc (*b*/*a* = 1.41, *h*/*b* = 0.167). **a** Averaged fcc lattice at 300 *s* after AOT substitution, calculated from a single big fcc grain. The fcc grain at this time is close to the perfect fcc lattice. **b**–**d** We select bcc nuclei with different sizes that are generated from the fcc grain in panel a and calculate their averaged lattices. As the size of bcc nuclei increases, the lattice structures change gradually from fcc to bcc. Significant changes will first be seen on *h*/*b*, caused by inter-layer adjusting. **e** At 8000 s after the AOT substitution, the fcc grain we investigate has mostly evolved into bcc solid while keeping the specific orientation relation. The bcc grain at this time will undergo further intra-layer adjustments to modify *b*/*a*. **f** The transition kinetics results in two specific orientation relations between the fcc mother lattice and bcc product lattice: Nishiyama–Wasserman (NW) and Kurdjumov–Sachs (KS) relations.

We note that all the nucleation pathways in Fig. 1e are diffusionless processes, and the produced bcc crystals have the same Nishiyama–Wassermann (NW) ((111)_fcc_∥(110)_bcc_, ${[\bar{2}11]}_{\text{fcc}}\parallel {[1\bar{1}0]}_{\text{bcc}}$) or Kurdjumov–Sachs (KS) ((111)_fcc_∥(110)_bcc_, ${[\bar{1}01]}_{\text{fcc}}\parallel {[\bar{1}1\bar{1}]}_{\text{bcc}}$) orientation relations with the parent fcc solid. We note that these two famous orientational relationships characterising the matching between the directions and planes of the parent fcc phase and those of the product bcc phase were established for martensitic transitions of steels (see, e.g. ref. ^4^). Moreover, we find twinnings of the product bcc grains inside one large parent fcc grain as commonly observed in hard materials (Fig. 3), which has NW and KS orientation relations with the parent fcc grain. Interestingly, we observe two sequential elementary steps to establish the orientational relation: first, the upper and lower layers of the fcc (111) plane slip relative to the middle layer (see the inter-layer sliding *h* from the left to middle panel of Fig. 1f), which mainly happens at the initial nucleation stage (see the evolution with respect to the nuclei size in Fig. 4). It is then followed by the two types of intra-layer deformation of the hexagonal plane at the growth stage, leading to the NW and KS relationships (see the two types of mode that change *b*/*a* in the most right panels of Fig. 1f). This is the first experimental illustration for the transformation kinetics at a microscopic level. Note that both the parent fcc (111) and final bcc (110) planes are parallel to our samples’ boundary wall. However, the orientation relation is established inside the sample already when the nucleus is small, suggesting that the specific orientation relation is a consequence of an intrinsic trend to reduce the diffusionless transformation’s energy cost, even for ultra-soft materials. We also note that the interface between the parent fcc phase and the product bcc phase in our soft colloidal systems is not as sharp as in hard materials (we will discuss this point in detail later).

For the two classes (athermal and thermally activated) of nucleation behaviours listed in Fig. 1e, we can further divide them into (i) inside-grain nucleation, (ii) grain boundary-assisted nucleation, and (iii) wall-assisted nucleation. In the following, we discuss them one by one, focusing on the parent lattice’s softness and how the system chooses the most appropriate pathway to overcome the nucleation barrier efficiently.

### Inside-grain nucleation

**Fig. 5:**
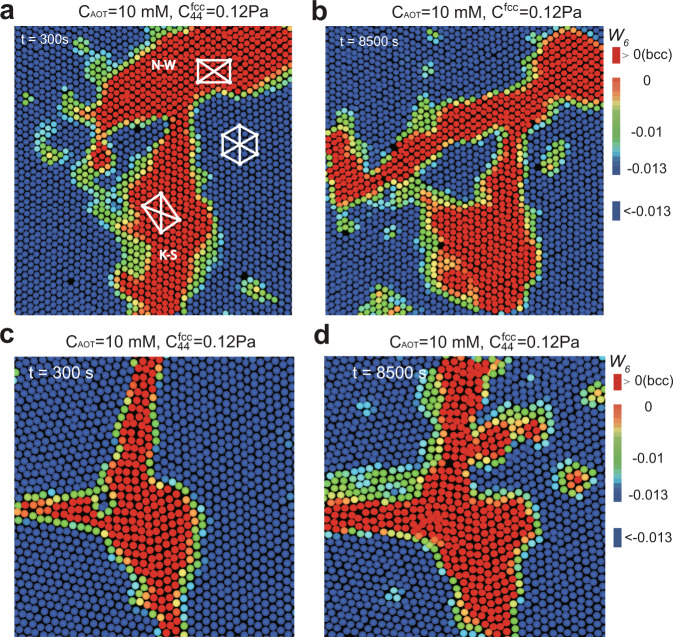
Pre-existing bcc nuclei inside the fcc grain and at the triple junction of grain boundaries. **a** Pre-existing bcc nuclei at the parent fcc grain at *C*_aot_ ~ 60 mM. They already have NW or KS orientation relation with the fcc lattice. **b** The little development of the bcc nuclei under the weak-driving force (*C*_aot_ ~ 10 mM) because of the absence of surface-energy and strain-energy reductions. **c** Pre-existing bcc nuclei at the triple junction of grain boundaries at *C*_aot_ ~ 60 mM. **d** The little development of the bcc nuclei under the weak-driving force (*C*_aot_ ~ 10 mM), because of the absence of significant surface-energy and strain-energy reductions.

First, we discuss the inside-grain nucleation (type (i)). For the low-driving-force case, classical models suggest the heterogeneous nucleation pathway characterised by nuclei pre-existing already above the transition temperature, stabilised by defects such as dislocations and stacking faults. This athermal pathway is selected when *E*_strain_ is much larger than *k*_B_*T* and dominant^1,7^. As expected, we find that there are pre-existing bcc nuclei at *C*_AOT_ ~ 60 mM (above the bcc-fcc phase line, *C*_AOT_ ~ 15 mM), and its growth is limited under the weak-driving-force (*C*_AOT_ ~ 10 mM, see Fig. 5a, b) because of the absence of surface- and strain-energy reduction in Eq. (1). Interestingly, thermal fluctuations cause the slow migration of the pre-existing bcc nuclei, as shown in Fig. 5b, which can be thought of as an interface roughening effect. We note that the flow induced in our samples by the AOT substitution (measured by a particle tracking of 20 s that is about ten times longer than the particles’ Brownian time) is negligible (<2 nm/s). Moreover, we do not find nucleation sites inside the parent fcc grain other than the pre-existing ones, indicating the absence of stress-driven nucleation induced by a sample flow. Together with the stress-driven nucleation in the presence of a sample flow reported in ref. ^14^, our observation highlights the significant role of stress on the martensitic transition under a low-driving force.

**Fig. 6:**
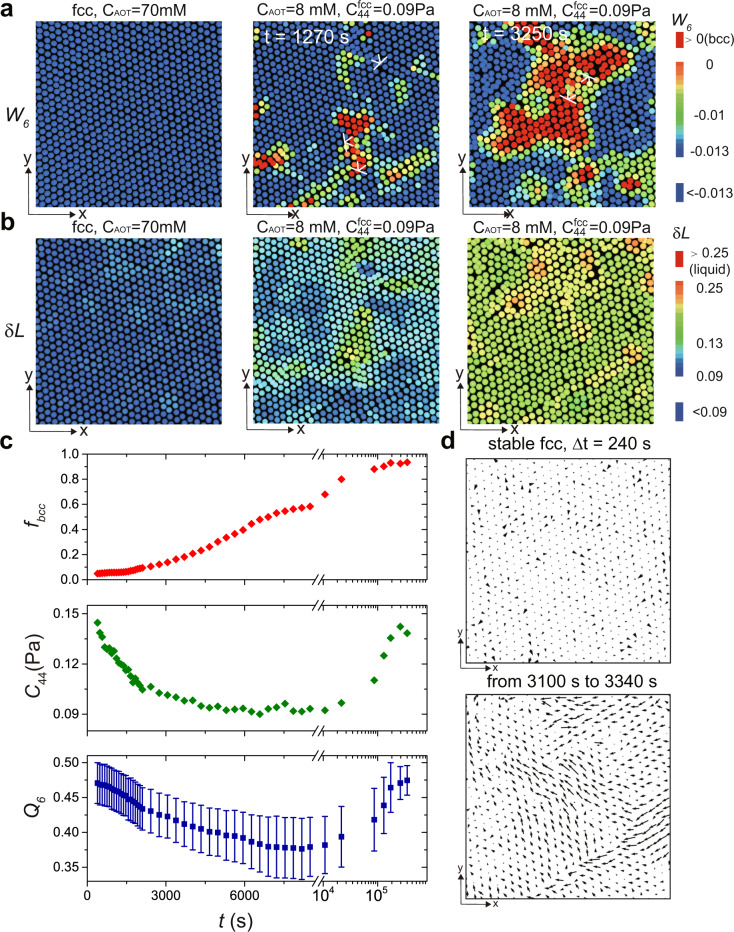
The thermally activated inside-grain homogeneous nucleation for low-modulus, strong-driving-force transition. **a** Colour plot of *W*_6_ of particles undergoing the fcc-to-bcc transition (red particles represent bcc; dislocations are specified). The bcc critical nucleus (*t* ~ 3250 s, *W*_6_ ≥ 0) appear in the region containing defects (*t* ~ 1270 s), which spontaneously forms in the defect-free fcc lattice. **b** The colour plot of the Lindemann parameter *δL* of particles undergoing the fcc-to-bcc transition, the regions with large Lindemann parameter (*t* ~ 1270 s) well correspond to the regions of large *W*_6_ fluctuations (small bcc nuclei) in **a**. **c** Time evolution of the fraction of bcc solids, *f*_bcc_, the component *C*_44_ of the shear modulus, and the average *Q*_6_ of the system. The error bar of *Q*_6_ represents the standard deviation. *C*_44_ before the truncation point is calculated for the parent fcc lattice, whereas the one after the truncation point is calculated for the product bcc lattice. *Q*_6_ is averaged over all particles in the system. In the nucleation stage (*t* < 3500 s), the formation of defects seen by the lowering of *Q*_6_ coincides well with the drop of the shear modulus *C*_44_. In the late growth stage (*t* > 7.5 × 10^4^ s), the slow eradication of defects seen by the growth of *Q*_6_ also coincides well with the increase of *C*_44_. **d** The displacement field of particles (at Δ*t* ~ 240 s) of the initial fcc lattice (see the top panel, *t* < 0) and that of particles after the formation of a critical bcc nucleus (*t* ~ 3250 s, bottom panel). Due to the small shear modulus ${C}_{44}^{{\rm{fcc}}}$, the bcc nuclei are created by displacive heterogeneities corresponding to the slipping motion of particles. The particles shown in panel d are the same as those in (**a** and **b**).

Surprisingly, bcc nuclei can be formed through thermal activation when the parent fcc lattice is soft enough. We can prepare defect-free grains without pre-existing bcc nucleus inside at *C*_AOT_ ~ 70 mM, which is above the fcc–bcc phase line and higher than 60 mM. At *C*_AOT_ ~ 8 mM (see the red arrow in Fig. 1b), we observe the thermally activated homogeneous nucleation kinetics inside dislocation-free grains, as illustrated in Figs. 6a, b and Supplementary Movies S1 and S2. Supplementary Movie S1 has a larger dimension (150 μm × 150 μm × 100 μm) but a lower time resolution (the time interval of ~300 s), whereas Supplementary Movie S2 has a lower dimension (150 μm × 150 μm × 20 μm) but a higher time resolution (the time interval of ~2.5 s). Large orange spheres in the movies represent bcc particles, whereas large blue spheres represent defects or their clusters (*Q*_6_ < 0.35). Note that particles with *Q*_6_ < 0.35 in soft colloidal systems are mostly highly distorted (defective) solid structures that contain a small amount of liquid-like (*Q*_6_ < 0.25) particles^41^.

Initially (at *C*_AOT_ ~ 70 mM), the parent fcc lattice is defect-free and stable. It is characterised by the negative value of *W*_6_ (*W*_6_ ~ −0.013) reflecting the fcc structure and also by its homogeneity due to the absence of defects (see the most left panel of Fig. 6a). The parent phase also has a small value of the Lindemann parameter, *δL*, of each particle, as shown in the most left panel of Fig. 6b. Then, after the strong-driving force towards the bcc phase is imposed by a jump of *C*_AOT_ to ~8 mM, defects spontaneously develop as fluctuations of the fcc lattice, which softens the nearby lattice sites and leads to the formation of small bcc nuclei at *t* = 1270 s (see the red particles in the middle panel of Fig. 6a). Correspondingly, the Lindemann parameter also increases around the defects (see the green region with larger *δL* in the middle panel of Fig. 6b). The spontaneous fluctuations keep growing and eventually lead to the formation of the bcc phase’s large nucleus (see the most right panels of Fig. 6a, b, *t* = 3250 s).

We show the temporal changes in the bcc solid fraction *f*_bcc_, the shear modulus component *C*_44_, and the structure order *Q*_6_, in the three panels in Fig. 6c, respectively, from top to bottom. They provide the following physical picture of the inside-grain homogeneous nucleation: The lowering of *C*_44_ and *Q*_6_ in the nucleation stage (*t* < 3500 s) generates heterogeneities in the displacement field in the parent lattice, triggering the nucleation of the product phase. This physical picture is strongly supported by the striking difference in the displacement field between the stable fcc lattice (the top panel of Fig. 6d) and the fcc crystal coexisting with the nucleated bcc crystal (the bottom panel of Fig. 6d).

**Fig. 7:**
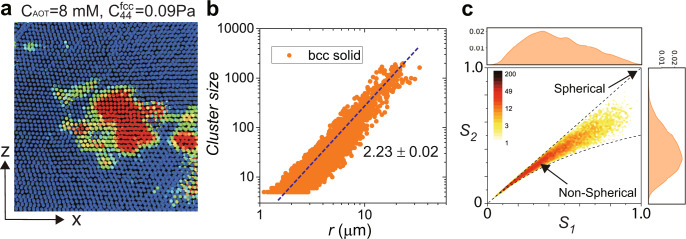
Anisotropic shape and ramified interface of in-grain nucleated bcc nuclei. **a** The *x*–*z* slice of in-grain nucleated bcc nuclei, where we can see the anisotropic shape with the ramified interface, similar to the one in the *x*–*y* slice (Fig. 6a). **b** The distribution of the mass-gyration radius in 3D for in-grain nucleated bcc nuclei. The fractal dimension of *d* ~ 2.23 manifests the ramified feature of the interface. **c** Density plot of bcc nuclei clusters on the shape-factor space (*S*_1_, *S*_2_), directly indicating their non-spherical shape. The point *S*_1_ = *S*_2_ = 1 represents a perfectly spherical shape. We can see that most bcc clusters belong to the non-spherical region. The most probable position is around (0.35, 0.3).

Surprisingly, we observe the increase of *C*_44_ and *Q*_6_ in the very late stage of bcc growth (Fig. 6c, at 20 h later), which reflects the slow eradication of the lattice defects that are created during the structural transformation. This behaviour again illustrates the critical role of lattice defects in the fcc-to-bcc transformation. Facilitated by thermally excited lattice defects, the in-grain bcc nuclei have a ramified interface (the most right panel of Fig. 6a) with anisotropy (Fig. 7), which should be a genuine feature of low-barrier solid-to-solid transition in the absence of applied stresses. This ramified interface produced by thermally activated nucleation distinctly contrasts with the sharp, twinning interface typically observed in metals.

The above finding reveals that nucleation pathways can be softness-dependent. It demonstrates the critical role of the thermal-excitation of defects for ultra-soft lattice in the nucleation process, which has not been reported before. This mechanism should be a new family member of solid-to-solid transitions characteristic to soft systems. Concerning this, it is worth mentioning that previous works in colloidal systems reported the liquid state-mediated nucleation kinetics, in which the nucleation involves the diffusive motion of particles without specific orientational relation to the parent phase^14,18,31^. The discovery of diffusionless defect-mediated spontaneous nucleation in addition to the diffusive one deepens our understanding of solid-solid transformations in soft, defect-free crystals.

### Grain boundary-assisted nucleation

It has been widely believed that grain boundaries (GBs) play pivotal roles in determining crystalline materials’ physical properties and kinetic behaviours^42–44^. For example, the triple junction of GBs has been widely recognised to be one of the most preferred nucleation sites in martensitic transitions of metals. As expected, we indeed find similar pre-existing bcc nuclei at the triple junction (*C*_AOT_ ~ 60 mM, see Fig. 5c, d). However, how GB assists the nucleation, e.g. whether through its roughening or via premelting caused by thermal activation, is poorly understood.

**Fig. 8:**
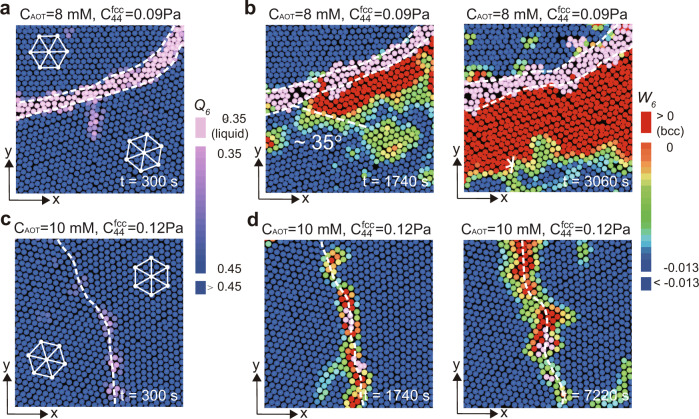
The grain boundary-assisted nucleation accompanied by premelting of the grain boundary. **a**, **b** Premelting of the grain boundary (*C*_AOT_ = 8 mM, *t* ~ 300 s after the AOT substitution) happens for the low-modulus transition in (**a**), leading to the heterogeneous nucleation initiating from it (*C*_AOT_ = 8 mM, *t* ~ 1740 s and *t* ~ 3060 s in (**b**), respectively). Bcc solid wets the liquid with an equilibrium contact angle of about 35°. The premelting also facilitates the displacive motion of the solid. **c**, **d** The premelting behaviour is absent at *C*_AOT_ = 10 mM, resulting in a small surface-energy reduction. Therefore, there is little bonus for the bcc nuclei generation and development.

Here, we show that the premelting of GBs greatly facilitates the grain boundary nucleation because it leads to a small bcc-liquid interface tension. As shown by the appearance of thick defective layers (*Q*_6_ < 0.35, pink colour) in Fig. 8a, the GB premelts under thermal fluctuations when the parent fcc lattice is soft enough (*C*_AOT_ ~ 8 mM, *t* = 300 s). Because the bcc-liquid interface tension, *γ*_bcc-liquid_, is smaller than the bcc-fcc one, *γ*_bcc-fcc_ (characterised by the contact angle of ~35° in left panel of Fig. 8b.), the premelting of GB helps to reduce the interface energy term *Aγ* in Eq. (1). The premelted layers can also facilitate the solid’s displacive movement, which can reduce the strain energy. Thus, we observe heterogeneous nucleation of bcc solid in the melt and its further growth (see the panels at *t* ~ 1740 s and *t* ~ 3060 s of Fig. 8b).

At *C*_AOT_ ~ 10 mM, on the other hand, GB does not premelt and only shows a roughening behaviour, as shown in Fig. 8c (*t* = 300 s). Thus, even after a much longer time (see the panels at *t* ~ 1740 s and *t* ~ 7220 s of Fig. 8d), the tiny bcc nuclei still stuck around the GB because the roughening behaviour causes a little surface-energy reduction in Eq. (1).

Under the strong-driving force at *C*_AOT_ ~ 8 mM, the mechanism of GB-assisted nucleation and growth accounts for about 70% of the bcc solids formation (see ‘Methods’ for the measurement details). This GB-assisted nucleation pathway we observed in soft colloidal crystals should be relevant to martensitic transitions close to the melting temperature, i.e. near the triple point at which the liquid and the two solid phases coexist, as long as the GB premelting can take place. This mechanism possibly paves the way for applying the long-existing GB engineering to the control of solid-to-solid transitions.

### Wall-assisted growth

For the weak-driving-force transition at *C*_AOT_ ~ 10 mM, the parent fcc lattice is not soft enough, and most GBs do not premelt; thus, the pre-existing bcc is the primary source of nucleation. In this case, the inside-grain and GB-assisted growth modes are not significantly facilitated. However, we find another pathway that can facilitate the growth of pre-existing nuclei: the wall-assisted growth, leading to the final bcc fraction of around 50%.

**Fig. 9:**
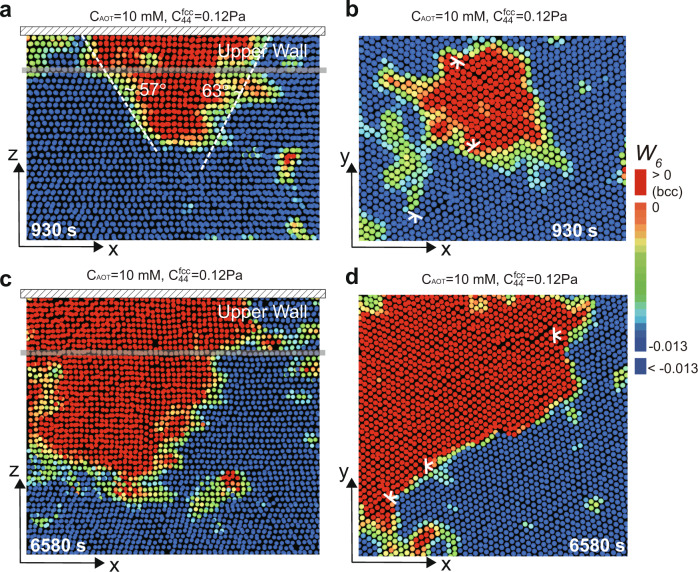
Wall-assisted growth for pre-existing nuclei observed at the weak-driving force. **a**, **b** Small pre-existing nuclei formed on the *x*–*z* and *x*–*y* planes of a wall at *t* = 930 s. The smooth wall wets the bcc solid with an equilibrium contact angle of about 60°. **c**, **d** The wall-assisted growth corresponding to (**a** and **b**), respectively, due to the surface-energy reduction of the wall-induced wetting.

The smooth flat wall has lower interface tension with bcc than fcc^21^, i.e. *γ*_bcc-wall_ < *γ*_fcc-wall_. We can confirm this by the ~60^∘^ wetting angle of a small bcc nucleus, as shown in Fig. 9a (*t* = 930 s). The wetting effect reduces the surface-energy term *Aγ* in Eq. (1), which promotes the further development of the bcc nucleus (Figs. 9c, d, at *t* = 6580 s).

**Fig. 10:**
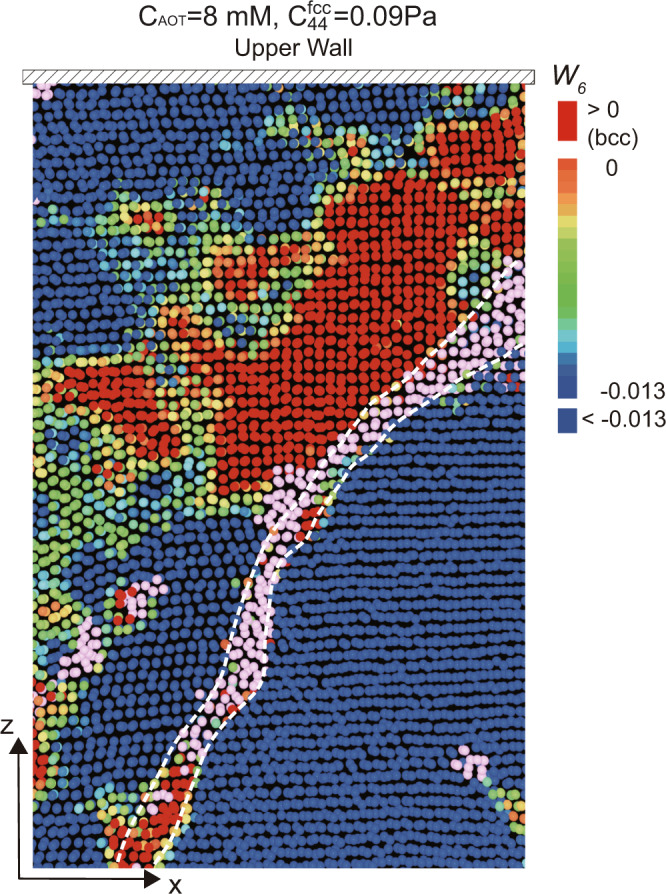
Rare realisation of wall-assisted growth for the strong-driving force. The *x*–*z* image suggests that wall-assisted growth is not favoured at the strong-driving force. Usually, the grain boundary ends at the wall, and the premelted grain boundary has a more predominant effect than the smooth wall. Accordingly, wall-assisted growth is rarely observed under a strong-driving force.

Now, a natural question arises: if the wall-bcc interface has smaller interface tension and the parent lattice is soft enough, does wall-assisted growth become the dominant mechanism? The answer is no. It is because that the GB-premelting has a more dominant effect than the smooth wall (Fig. 10). Usually, the grain boundary ends at the system’s boundary wall, and the grain boundary-assisted growth accompanied by premelting has a lower surface energy than the wall-induced growth, because $({\gamma }_{\text{bcc-wall}}-{\gamma }_{\text{fcc-wall}})/\cos {\theta }_{1} \sim {\gamma }_{\text{bcc-fcc}} \sim ({\gamma }_{\text{bcc-liquid}}-{\gamma }_{\text{fcc-liquid}})/\cos {\theta }_{2}$ (*θ*_1_ ~ 60^∘^ in Fig. 9a and *θ*_2_ ~ 35^∘^ in Fig. 8b). Thus, wall-induced growth rarely happens when GB-premelting is present. On the other hand, when GB does not premelt, it can effectively be regarded as a rough wall, so that wall-assisted growth on a smooth flat wall should be more preferred.

## Discussions and outlook

In addition to the non-thermal nucleation pathways widely observed in hard materials, the martensitic transition kinetics of soft colloidal crystals has revealed the previously unknown nucleation pathways, such as the thermally activated in-grain nucleation, GB-premelting-assisted nucleation, and wall-assisted growth. We discuss the generality of these results for each of these three pathways, focusing on how the energy barrier is reduced, reflecting the system’s character.

For the in-grain homogeneous nucleation, the low shear modulus and the absence of liquid intermediates suggest that the parent lattice is likely near the mechanical instability point without exceeding its melting point. In other words, a weak superheating ability, where the superheating limit of the system is located above but very close to the melting point, should be the critical requirement for this type of solid-solid transformation. More specifically, we assume that at least one of the shear modulus components becomes zero at the superheating limit of a solid state, leading to the melting of the solid into a liquid barrierlessly. Thus, this nucleation pathway requires a weak superheating ability as well as a suitably soft lattice modulus before melting. With a weak superheating ability, defects are thermally excited frequently near the melting point, and they serve as the primary source of nucleation sites. We note that the superheating ability is closely related to the potential softness and density of the system, as well known. From these considerations, we expect that the in-grain homogeneous nucleation possibly exists in systems with soft and relatively long-range overlapped potentials (e.g. charge-, dipole- and quadrupole-related interactions), solution-based materials (such as polyelectrolyte and charged proteins), and highly compressed soft polymers.

In the GB-premelting-assisted nucleation, a small surface-energy term in the barrier plays a critical role. Because thermal-excitation (rather than applying stress) is still the reason for this nucleation pathway, a lower modulus of the parent lattice and a higher driving force of the solid-to-solid transition reducing the nucleation barrier could also facilitate this process. In this nucleation pathway, the GB-premelting triggers the generation of small nuclei, and their growth is controlled by the balance between the surface energy, strain energy, and chemical-potential differences. Accordingly, a weak superheating ability is unnecessary for this pathway; instead, the GB-premelting alone is primarily responsible. We expect this mechanism to be generally realised because the increase in temperature could trigger the GB premelting before the bulk melting happens. Accordingly, besides soft systems with a weak superheating ability, such a kinetic pathway is likely to be seen in a broad class of materials with a high-temperature triple point (of a liquid and two solid states)^45,46^. We expect that this pathway is operative when the pressure or temperature is close to the triple point, which can happen, e.g. in some organic materials.

The wall-induced growth is similar to the GB-premelting-assisted nucleation in the sense that the reduction of the surface-energy term in the barrier plays a crucial role. So, a weak superheating ability is not a necessary condition for this pathway. Moreover, a lower modulus of the parent lattice and a higher driving force of the solid-to-solid transition also help facilitate this pathway. Accordingly, this pathway is also likely to be seen in the systems mentioned above for the GB-premelting-assisted nucleation.

In these three pathways found in our soft colloidal systems, we observe interesting morphologies of the child bcc phase. Firstly, inside one grain of the parent solid, we find nearly 70° twinning grain boundary of the child bcc phase, which have NW and KS relationships with the parent solid, respectively. Such a morphology during the transition obviously reduces the interface energy between the parent phase and the child phase (two bcc grains that have the same NW or KS relationship with the parent solid could form a grain boundary that is less close to a coherent state, Fig. 3). Next, the interface is not as sharp as in hard materials, which may be regarded as an interface roughening effect due to the low modulus of soft systems. Moreover, the grain boundary networks (large-angle grain boundaries and triple junctions) in the parent phase are retained during the transition, which also reduces the interface energy cost of the transition.

We hope that these kinetic pathways and morphologies found in soft materials deepen the theoretical understanding of the physical mechanism of solid-to-solid transformations and pave the way to control the diffusionless solid-solid transformations, which should be broadly applicable to crystals of numerous materials.

## Methods

### Samples

We suspend DiIC_18_ dyed and poly hydroxystearic acid polymers grafted poly(methyl methacrylate) (PMMA) colloids (diameter *σ* = 1.8 μm, polydispersity ~ 2.5%, https://www2.ph.ed.ac.uk/~abs/) in a mixture of the non-polar solvents (tetrachloroethylene +hexachlorobutadiene + decahydronaphthalene, Aldrich), whose refractive index and density are precisely matched with those of particles. The addition of AOT (sodium di-2-ethylhexyl sulfosuccinate, Aldrich) into the colloidal suspensions produces ionic AOT micelles that can change the interparticle potential^36,37^. The PMMA particles are negatively charged in our system and interact with each other via weakly screened Coulomb repulsion, $u(r)=\alpha \exp [-\kappa \sigma (r/\sigma -1)]/(r/\sigma )$ for *r* > *σ*, with 1/*κ* being the Debye screening length and *α* being the effective interaction strength in the suspension that can be adjusted by the AOT concentration^36,37^. By reducing the AOT concentration at a constant particle volume fraction *ϕ*, we can change the stable solid structures from fcc to bcc, as shown by the phase diagram in Fig. 1b.

### Special sample cell

We design a new sample cell (length = 6 mm, width = 1.5 mm, depth = 0.1mm) with a semi-permeable membrane (Fig. 1a)^47^. The bottom of the cell is made of a cover glass, allowing confocal microscopy observation through it. The particle cell containing the PMMA suspension is separated from the large reservoir containing AOT solutions by a semi-permeable membrane with a pore size of 0.22 μm. Ionic AOT micelles (*r* ~ 1 nm) can pass through the membrane, whereas PMMA particles (*σ* ~ 1.8 μm) cannot. Thus, by adjusting the AOT concentration *C*_AOT_ in the reservoir, we can adjust the screening length of the repulsive Coulomb potential between particles in the sample cell through fast ion diffusion. We estimate the characteristic diffusion time *τ* of AOT micelles over a distance *h* through the membrane to be *τ* ~ 10 s, from *τ* ~ 6*πηh*^2^*a*/*k*_B_*T*, where *η* is the viscosity of the solvents (1.3 cP), *a* is the hydrodynamic radius of AOT micelles (~1 nm) and *h* is the depth of sample cells (~100 μm). We stress that this diffusion time is much shorter than the transition time, and thus, the substitution can be regarded as almost instantaneous. We have also confirmed that when the system is equilibrated after the substitution, there is no noticeable concentration gradient in the system. This conclusion is based on the experimental observations that there are no noticeable differences between the behaviours at the top and bottom of the sample cell. We have also estimated the flow rate from tracking the particle motion in a 2D horizontal plane to be less than 2 nm/s, indicating that our protocol is non-perturbative^14^. This special setup allows us to trigger and observe fcc-to-bcc martensitic transitions in situ at the single-particle level in real-time with 3D confocal microscopy.

### Experimental protocol

We first prepare parent fcc crystals at a high *C*_AOT_. At *C*_AOT_ ~ 60 mM (*ϕ* ~ 22%), we can find a small amount of pre-existing bcc solid at the triple junction of grain boundaries, the sample walls, and inside the fcc grains. At *C*_AOT_ ~ 70 mM (*ϕ* ~ 22%), the pre-existing bcc becomes less frequent, and we can find defect-free fcc grains. Then, we replace the AOT solution in the reservoir by the low concentration one, indicated by the orange arrow in Fig. 1b (*C*_AOT_ ~ 10 mM, *ϕ* ~ 20%, bcc-stable). It causes a quick (~10 s) reduction of the AOT concentration in the sample cell, triggering the fcc-to-bcc martensitic transition. We observe the transition process in-situ by a Leica SP8 fast confocal microscope (~10 μm/s along *z* direction, with a 150 × 150*μ*m^2^ *x*–*y* view field). We recorded both thick regions (about 150 μm × 150 μm × 100 μm, including ~ 250,000 particles) with lower time resolution (~300 s) and thin regions (about 150 μm × 150 μm × 20 μm, including ~ 50,000 particles) with higher time resolution ( ~2.5 s) for analysis. The field of view with the confocal microscope objective is about 150 μm × 150 μm, whereas the typical grain size in (*x*, *y*) plane is about 100–300 μm. To avoid the bias of the sampling, we choose two working modes of a motorised scanning stage of our confocal microscope to make the large-field-of-view sampling: the large-filed-view modes (a 4 × 4 area that is much larger than the grain size) and the tile scanning mode (randomly pick up 16 regions). The confocal microscope can automatically do the 3d scanning of each sub-region after we make the setup. The fractions of 70% and 30% indicated in the second column of Fig. 1e are estimated by the relative amounts of bcc crystallites formed through the GB-assisted pathway and in-grain nucleation, respectively, at the timing when the total bcc fraction is about 40–50%. Because the final bcc crystallites formed through the two pathways will finally merge into large bcc grains (reaching nearly 90%) under a high driving force, it is hard to discriminate their contributions from each other. On the other hand, the bcc crystals from the in-grain nucleation and GB-assisted nucleation can be distinguished in the growth stage when the total bcc fraction is about 40–50%.

### Structural analysis

We capture time-resolved 3D images and perform 3D particle tracking, using IDL^48^ (http://www.physics.emory.edu/faculty/weeks//idl/index.html) and Trackpy^49^ (http://soft-matter.github.io/trackpy/v0.5.0/) to extract all particles’ positions as a function of time, making the subsequent structural analysis possible. In this work, we use the two most frequently used structural order parameters, which are coarse-grained bond orientational order parameters, *W*_6_ and *Q*_6_ (see below for their definitions). *W*_6_ is used to distinguish fcc and bcc by its sign (*W*_6_ ≥ 0 for bcc and *W*_6_ < 0 for fcc), whereas *Q*_6_ quantifies the degree of crystalline order of the solid (particles with *Q*_6_ < 0.35 are regarded as defective structures, which mainly consist of highly distorted (defective) solid and a amall amount of liquid-like (*Q*_6_ < 0.25) particles.)^21,22,39–41^ (Fig. 2).

### Definition of order parameters, *Q*_6_, *W*_6_ and *δL*

First, we calculate the local bond orientational order as ${q}_{l,m}(i)=\mathop{\sum}\limits_{f\in F(i)}\frac{A(f)}{A}{Y}_{l,m}({\theta }_{i,j},{\phi }_{i.j})$, where *A*(*f*) is the surface area of the Voronoi cell facet *f* separating centre particle *i* and its neighbour *j*, and *A* is the total surface area of the Voronoi cell boundary *F*(*i*), *Y*_*l*,*m*_(*θ*_*i*,*j*_, *ϕ*_*i*,*j*_) are the spherical harmonics with *m* ∈ [−*l*, *l*], *θ*_*i*,*j*_ and *ϕ*_*i*,*j*_ are the polar and azimuthal angles of the vector **r**_*ij*_ = **r**_*i*_ − **r**_*j*_, where **r**_*i*_ is the position vector of particle *i* and **r**_*i*_ is the position vector of its neighbouring particle *j*. The coarse-graining version is calculated as ${Q}_{l,m}=\frac{1}{Nb}\mathop{\sum }\nolimits_{k = 0}^{Nb}{q}_{l,m}(k)$, with *Nb* being the number of neighbours determined by Voronoi cell analysis. Then, we obtain the rotationally invariant coarse-grained order parameters of particle *i*, *Q*_*l*_(*i*) and *W*_*l*_(*i*), as follows: ${Q}_{l}(i)={(\frac{4\pi }{2l+1}\mathop{\sum }\nolimits_{m = -l}^{l}{\left|{Q}_{l,m}(i)\right|}^{2})}^{1/2}$ and $${W}_{l}(i)=\mathop{\sum }\nolimits_{{m}_{1}+{m}_{2}+{m}_{3} = 0}^{l}\left(\begin{array}{lll}l&l&l\\ {m}_{1}&{m}_{2}&{m}_{3}\end{array}\right)\frac{{Q}_{l,{m}_{1}}(i){Q}_{l,{m}_{2}}(i){Q}_{l,{m}_{3}}(i)}{{(\mathop{\sum }\nolimits_{m = -l}^{l}{\left|{Q}_{l,m}(i)\right|}^{2})}^{3/2}}$$.

We also introduce the Lindemann parameter *δL*^41^. Here, *δL* = *δ*/*a*, where *δ*^2^ is the height of the time-independent plateau of the mean-square displacement 〈Δ**r**^2^(*t*)〉 and *a* is the lattice constant. In this work, it is calculated from −10 s to +10 s for each particle.

### Calculation of shear modulus *C*_44_

The shear modulus is calculated, assuming the elastic interaction between particles and the Boltzmann distribution for the interparticle bond length^41^. We impose a shear matrix *ϵ* on bcc or fcc lattice matrix, then expand the strain energy *E* into the combination of the spring constant *k* and *ϵ*. Then, $4{V}_{0}{C}_{44}=\frac{{d}^{2}E}{d{\epsilon }^{2}}$. For bcc lattice, we have ${C}_{44}=\frac{{k}_{1}}{a}+\frac{2{k}_{3}}{a}$. For fcc lattice, on the other hand, we have ${C}_{44}=\frac{{k}_{1}}{a}+\frac{2{k}_{2}}{3a}$^50^. Here *a* is the lattice constant, and *k*_1_, *k*_2_, and *k*_3_ are the spring constants for nearest-neighbour, second-nearest-neighbour and third-nearest-neighbour bonds, respectively. We determine *a* from the 3D pair correlation functions. We measure each spring constant by determining the distribution of the corresponding bond length *l*_*i*_, and assume that it obeys the equilibrium Boltzmann distribution as

2 $$P({l}_{i})={A}_{0}{\exp }^{\frac{-{E}_{\text{elastic}}}{{k}_{{\mathrm{B}}}T}}={A}_{0}{\exp }^{\frac{{k}_{i}{(x-{l}_{i})}^{2}}{2{k}_{{\mathrm{B}}}T}}={A}_{0}{\exp }^{\frac{{(x-{A}_{1})}^{2}}{2{A}_{2}^{2}}}.$$

We can experimentally get the neighbour bond length distribution at different time, and fit it with the last expression to get *A*_0_, *A*_1_, and *A*_2_. Then, the elastic constant *k* and average bond length *l* are obtained as

3 $${k}_{i}=\frac{{k}_{{\mathrm{B}}}T}{{A}_{2}^{2}}\qquad l={A}_{1}.$$

### Shape-factor calculation using inertial tensor

For a quantitative description of bcc nuclei’s shape, we take the following steps to define the shape-factor parameters using the inertial tensor. The inertial tensor of a cluster of particles with *N* neighbours is defined as^51^:

4 $$\bar{I}=\mathop{\sum }\limits_{i = 1}^{N}({\overrightarrow{r}}_{i}\cdot {\overrightarrow{r}}_{i})\bar{1}-{\overrightarrow{r}}_{i}\bigotimes {\overrightarrow{r}}_{i},$$

where ${\overrightarrow{r}}_{i}$ is the vector from the centre of mass of the cluster to particle *i*, $\bar{1}$ is the unit tensor, and ⨂ denotes tensor product. Then, we calculate the three eigenvalues of $\bar{I}$, *v*_1_, *v*_2_, and *v*_3_, from the largest to smallest. Then, we define *S*_1_ = *v*_3_/*v*_2_ and *S*_2_ = *v*_3_/*v*_1_. Points on the *S*_1_ − *S*_2_ space can characterise the cluster anisotropy.

## Supplementary information

Supplementary Movie 1

Supplementary Movie 2

Description of Additional Supplementary Files

## Data Availability

The experimental data that support the findings of this study are available within the article.

## Code availability

The codes associated with this manuscript are available from the corresponding author upon request.
