# Supplementary material for: Revealing thermally-activated nucleation pathways of diffusionless solid-to-solid transition
Source: Nat Commun. 2021 Jun 30;12:4042. doi: 10.1038/s41467-021-24256-9 (PMC8245452; doi:10.1038/s41467-021-24256-9)
Supplement: Supplementary file 3 — Description of Additional Supplementary Files [file 41467_2021_24256_MOESM3_ESM.pdf]

Title: Supplementary Movie 1

Description: The process of thermally-activated homogeneous nucleation inside dislocation-free grains corresponding to Figs. 6a and b. This movie covers a volume of 150 μm$\times$150 μm$\times$100 μm) with the time interval of $\sim$300 s. Large orange spheres represent bcc particles, whereas large blue spheres represent defects or their clusters ($Q_6 < 0.35$).

Title: Supplementary Movie 2

Description: The same as Movie S1 but with a smaller volume (150 μm$\times$150 μm$\times$ 20 μm) and a higher time resolution (the time interval of $\sim$2.5 s).
